# The neuro-psychological dimensions of piano interaction: from mental health therapy to intelligent systems

**DOI:** 10.3389/fpsyg.2026.1725791

**Published:** 2026-07-07

**Authors:** Lijuan Cui

**Affiliations:** Department of Modern Music, Shanxi Vocational College of Art, Taiyuan, Shanxi, China

**Keywords:** auditory-motor integration, cognitive rehabilitation, human-computer interaction, mental health, neuroplasticity, piano-based intervention

## Abstract

This article synthesizes contemporary research on the multifaceted impacts of piano interaction on human cognition, mental health, and neural processes, and explores its translation into intelligent human-computer systems. Evidence from clinical psychology demonstrates that structured piano training can significantly alleviate anxiety and depression in the elderly, enhance executive functions and working memory in aging populations, and serve as an effective component within multi-element interventions for severe mental illness, as exemplified by the GET UP PIANO trial. Neuroscientific investigations reveal that these benefits are supported by a specialized auditory-motor network, encompassing premotor and parietal cortices, which exhibits significant plasticity in experts and is engaged during both music perception and mental imagery. Unique patterns of musical processing in special populations, such as preserved fronto-temporal connectivity for song in autism spectrum disorder, provide a neurobiological rationale for music-based therapies. Critically, these findings are now informing the development of intelligent technologies. We explore how principles of cross-modal correspondence and personalization are being leveraged to create adaptive systems for health-tech, including closed-loop neurofeedback for neuromodulation, smart keyboards for motor rehabilitation using biofeedback, and AI-driven analysis of musical improvisation for mental health assessment. Finally, the review addresses key challenges, including the need for methodological standardization, mechanistic elucidation, and ethical HCI design focused on data security and long-term engagement. The convergence of neuroscience, clinical practice, and technology positions the piano as a powerful and evolving interface for enhancing human health and cognitive resilience.

## 1 Introduction

The piano is far more than a musical instrument; it is a sophisticated biomechanical and cognitive interface that demands the integrated engagement of multiple neural systems in real-time. Performing on the piano requires the translation of abstract visual symbols (sheet music) into precise, bimanual motor sequences, all while the auditory system provides continuous feedback for error correction and expressive tuning. This complex sensorimotor loop, engaging auditory, motor, visual, and emotional systems simultaneously, makes piano training a powerful vehicle for neuroplastic change. Neuroscientific evidence confirms this, demonstrating that piano practice activates a widespread network including the dorsal premotor cortex, pre-SMA, and intraparietal sulcus, which are critical for sensorimotor integration and sequence learning (Brown et al., 2013). This intricate coordination is not merely for artistic expression; it forms the basis for therapeutic applications. For instance, the mental simulation of physical exercise, a form of enactive cognition similar to mentally practicing a piece, has been shown to engage broad brain networks, including the insular cortex, highlighting the brain’s capacity for detailed action simulation (Philips et al., 2024). The piano, therefore, serves as a unique tool to access and modulate brain function, making it an ideal platform for interventions targeting cognitive enhancement, emotional regulation, and motor rehabilitation.

The use of music for healing has ancient roots, but its application in modern clinical practice has evolved from a holistic, often intuitive practice to an evidence-based discipline grounded in empirical research. Early work often focused on the receptive listening experience. However, the modern paradigm has shifted toward active, structured musical participation, with piano-based interventions standing at the forefront. This shift is exemplified by the development of structured protocols, such as Individualized Piano Instruction (IPI) for older adults, which demonstrated measurable improvements in executive functioning and working memory, providing some of the first controlled evidence for its cognitive benefits (Bugos et al., 2007). Furthermore, large-scale, pragmatic clinical trials have established the feasibility and effectiveness of complex psychosocial interventions that can include musical elements, setting a new standard for evidence-based practice. This progression from anecdotal use to rigorous, hypothesis-driven research underscores the maturation of the field and provides a solid theoretical and methodological foundation for contemporary studies.

This review aims to synthesize the growing body of literature on the neuro-psychological dimensions of piano interaction, traversing a path from clinical outcomes to underlying neural mechanisms and, finally, to technological translation (Table 1). We will first consolidate evidence on the therapeutic efficacy of piano-based interventions across a spectrum of conditions, including anxiety and depression in the elderly (Song and Guan, 2025), cognitive decline (Bugos et al., 2007; James et al., 2020), and severe mental illness (Ruggeri et al., 2015). Subsequently, we will delve into the neural orchestration that underpins these benefits, exploring the auditory-motor network in experts and novices (Landau and D’Esposito, 2006; Sluming et al., 2007), the neural basis of music perception in special populations like autism spectrum disorder (Sharda et al., 2015), and the cognitive processes involved in musical imagery and enaction (Martin et al., 2018; Philips et al., 2024). Finally, and crucially, this review will explore how these neuroscientific and clinical principles might 1 day be translated into intelligent human-computer systems. We will examine how insights into cross-modal correspondences (Salgado-Montejo et al., 2016), user state modeling (O’Toole et al., 2021), and neural decoding (Carrillo-Reid and Yuste, 2020) could inform the conceptual development of adaptive technologies for rehabilitation, mental health, and education. It is important to note that the systems discussed in section 5 are largely speculative at present, representing a translational vision grounded in current research rather than established clinical or educational practice. This section aims to outline potential future directions while clearly distinguishing between evidence-based principles and the conceptual applications they might 1 day inspire.

**TABLE 1:** Summary of key clinical studies on piano-based interventions.

Study	Population	Intervention type	Primary outcomes	Key findings
Song and Guan (2025)	Elderly individuals (60–70 years)	Group piano lessons (2x/week)	Anxiety (Taylor scale); depression (beck inventory); Wellbeing (WAM)	- Anxiety scores decreased by > 50%- depression scores halved- significant improvement in wellbeing, activity, and mood
Bugos et al. (2007)	Healthy older adults	Individualized piano instruction (IPI)	Executive function (trail making test; digit symbol)	- Significant improvement in executive functioning and processing speed vs. control- demonstrated transfer from musical training to non-musical cognitive domains
Ruggeri et al. (2015) GET UP piano	First-episode psychosis (FEP) patients	Multi-element psychosocial intervention (CBT, family intervention, case management)	Symptom severity (PANSS); global functioning; hospitalization	- Feasible to implement in routine care- greater reduction in symptom severity and improvement in global functioning vs. treatment as usual (TAU)
James et al. (2020) TBM trial	Healthy retired elderly	Piano instruction vs. musical listening (RCT)	Cognitive function; brain plasticity (fMRI, DTI)	- A longitudinal protocol investigating neural mechanisms of cognitive benefits- Aims to link behavioral change to functional and structural brain plasticity

## 2 Methodology

This review aims to synthesize the growing body of literature on the neuro-psychological dimensions of piano interaction, traversing a path from clinical outcomes to underlying neural mechanisms and, finally, to technological translation. Given the interdisciplinary nature of this topic and the heterogeneity of the available literature, a narrative review approach was adopted to provide a comprehensive overview while allowing for the integration of diverse study types, including randomized controlled trials, observational studies, neuroimaging investigations, and preliminary reports.

**FIGURE 1:**
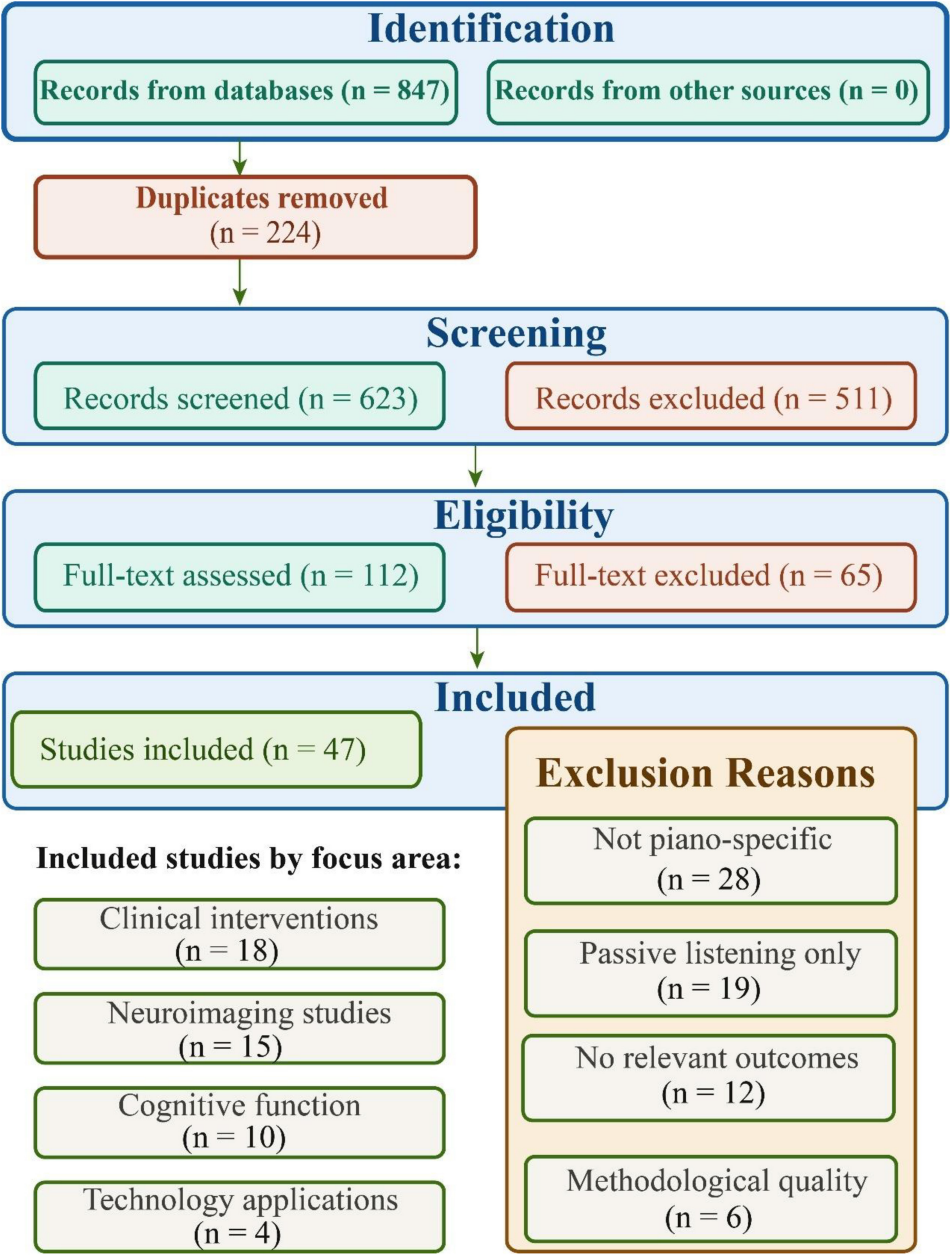
PRISMA chart flow for the investigation to draw this study.

To identify relevant publications, a systematic search of the electronic databases PubMed, Web of Science, and PsycINFO was conducted. The inclusion and exclusion of articles searched has been provided in PRISMA flow chart (Figure 1). The search strategy combined terms related to the intervention (“piano,” “keyboard,” “music therapy,” “musical training”), the outcomes of interest (“mental health,” “cognition,” “depression,” “anxiety,” “neuroplasticity,” “motor rehabilitation”), and the populations studied (“aging,” “elderly,” “autism,” “schizophrenia,” “stroke”). The reference lists of key articles were also hand-searched to identify additional relevant studies. The search was limited to English-language publications, with no restriction on publication date to capture both foundational and contemporary work. Studies were included if they: (1) involved piano playing, keyboard training, or music therapy incorporating piano as a primary instrument; (2) reported outcomes related to mental health, cognitive function, neural mechanisms, or motor rehabilitation; (3) were published in peer-reviewed journals or as part of established scientific conference proceedings. Given the exploratory nature of this review, no studies were excluded based on design or sample size; however, the level of evidence is explicitly considered throughout the discussion. Meeting abstracts and preliminary reports are clearly identified as such, and strong efficacy claims are avoided when based on limited evidence. Studies focused exclusively on other instruments or on passive music listening without active piano engagement were excluded unless they provided essential mechanistic or comparative insights.

**FIGURE 2:**
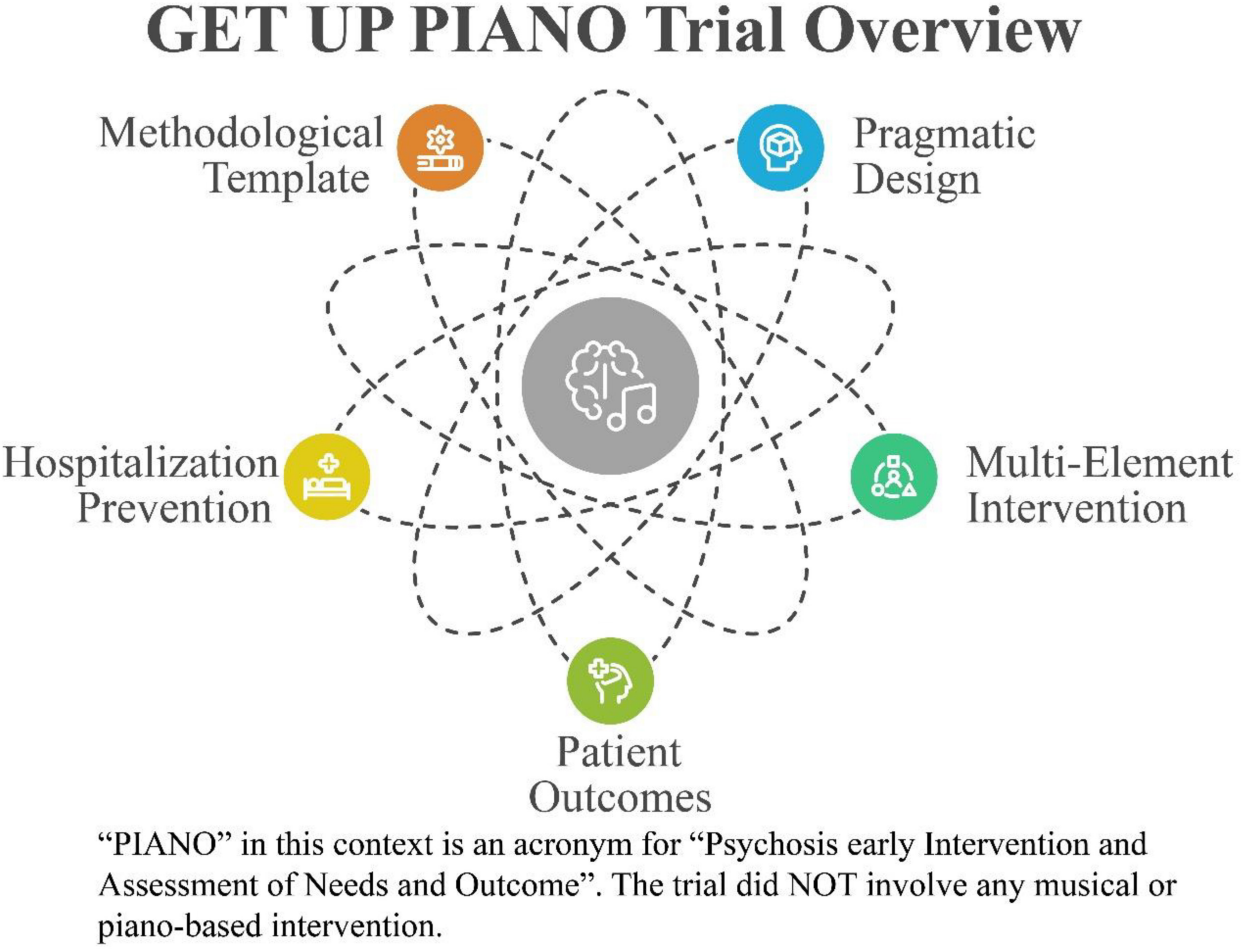
GET UP Piano trial overview to discuss psychosis early intervention and assessment of needs and outcome.

The GET UP PIANO trial (Ruggeri et al., 2015, 2012), a landmark cluster-randomized controlled study conducted across 117 community mental health centers in Italy, demonstrated the feasibility and effectiveness of a multi-element psychosocial intervention for first-episode psychosis. As the acronym indicates, “PIANO” here refers to “Psychosis early Intervention and Assessment of Needs and Outcome,” and the trial did not involve any musical component (Figure 2). This study is discussed in the present review not as evidence for piano-based therapy *per se*, but rather as an exemplary model of rigorous, pragmatic trial methodology that could be adapted to evaluate genuine piano interventions. The distinction is critical: while the GET UP PIANO trial provides a template for how complex interventions can be assessed in “real-world” settings, the therapeutic benefits of actual piano training must be evaluated on their own terms, through studies such as those by Song and Guan (2025) and Bugos et al. (2007), which directly investigate structured piano instruction.

## 3 Piano-based interventions in mental health and cognitive rehabilitation

This section consolidates findings on the therapeutic and cognitive benefits of piano engagement across different populations.

### 3.1 Alleviating anxiety and depression

Piano-based interventions have demonstrated significant efficacy in alleviating symptoms of anxiety and depression across diverse populations, establishing the instrument as a valuable tool in the non-pharmacological therapeutic arsenal. The most direct evidence comes from a recent study by Song and Guan (2025), which investigated the impact of a structured piano course on the mental health of elderly individuals. Following a 6-month regimen of bi-weekly lessons, participants exhibited a 19-point decrease on the Taylor anxiety scale (representing a > 50% reduction from baseline) and a 21.5-point decrease on the Beck Depression Inventory (BDI). While these reductions appear substantial, the absence of reported effect sizes (e.g., Cohen’s *d*) and the lack of established Minimal Clinically Important Difference (MCID) thresholds for the Taylor scale in elderly populations limit definitive interpretation of clinical significance. For the BDI-II, established MCID thresholds suggest that a reduction of approximately 17.5% from baseline (or 3–5 points) represents the smallest change patients perceive as beneficial, with more severely depressed or treatment-resistant populations requiring larger reductions (approximately 32%) to report meaningful improvement (Button, 2015). The observed 21.5-point reduction in Song and Guan’s study would substantially exceed these MCID thresholds, suggesting clinically meaningful improvement, though this interpretation must be tempered by the study’s small sample (*n* = 30) and absence of control group or effect size reporting. This aligns with other clinical observations, such as the use of piano performance as an intervention for patients with bipolar disorder (Liu, 2024) and anxiety disorders (Zhang, 2025), suggesting a transdiagnostic potential for music-making. The therapeutic mechanism is twofold. While these preliminary reports have suggested potential applications for piano-based interventions in anxiety disorders (Zhang, 2025), these findings are currently only available as meeting abstracts and should be interpreted with caution.

### 3.2 Enhancing cognitive function in aging and neurodegeneration

The potential of piano training to serve as a robust cognitive intervention for older adults is supported by empirical evidence, though effect sizes are modest and clinical significance requires careful interpretation. A foundational study in this area was conducted by Bugos et al. (2007), who implemented an Individualized Piano Instruction (IPI) protocol for musically naïve older adults. In this randomized controlled trial, the experimental group that received 6 months of personalized piano lessons demonstrated significant improvements on neuropsychological assessments compared to a no-treatment control group. Specifically, they showed enhanced performance on the Trail Making Test and the Digit Symbol Substitution Test (DSST), which are key measures of executive functions, processing speed, and cognitive flexibility. However, Bugos et al. (2007) did not report effect sizes, and subsequent replications with larger samples have found small effect sizes (Cohen’s *d* ≈ 0.20–0.24) for cognitive outcomes, with benefits most pronounced among individuals with mild cognitive impairment (Lister et al., 2023).

Regarding clinical significance, established MCID thresholds for the DSST in older adults who fall suggest that improvements of ≥ 2–3 symbols represent meaningful change (Jehu et al., 2021). For the Trail Making Test, no established MCID exists, though distribution-based estimates suggest that reductions of approximately 50.5 seconds on Trail Making Test B-A may indicate clinically meaningful improvement (Jehu et al., 2021). Without these metrics, the magnitude of benefit from Bugos et al. (2007) intervention remains difficult to quantify. This study provided some of the first controlled evidence that learning a complex skill like the piano in later life can induce transfer effects, mitigating age-related cognitive decline in domains critical for independent living.

Building upon these findings, contemporary research is now investigating the underlying neurobiological mechanisms of such cognitive benefits through large-scale, longitudinal studies. The ongoing “Train the Brain with Music (TBM)” trial, described by James et al. (2020), represents a cutting-edge example of this approach. This two-site, randomized controlled trial is comparing the effects of 1 year of protocolled piano instruction against an active control group receiving musical listening awareness in a cohort of 155 healthy retired elderly individuals. The TBM trial is particularly significant because it combines comprehensive cognitive and perceptual-motor testing with multi-modal neuroimaging (functional and structural MRI) and blood sampling at multiple time points. By tracking changes over 12 months of training and a subsequent 6-month follow-up, it aims to directly link behavioral improvements in working memory and executive functions to functional and structural brain plasticity in regions such as the prefrontal cortex, temporal areas, and the basal ganglia. The results of this rigorous study are poised to provide an unprecedented understanding of how musical practice can stimulate neuroplasticity and offer a compelling, intrinsically motivating strategy for promoting cognitive resilience in an aging global population.

### 3.3 Applications in severe mental illness

For severe mental illness, multi-element psychosocial interventions have demonstrated efficacy in pragmatic trials (see Methodological Sidebar regarding the GET UP PIANO trial as a model for rigorous evaluation in real-world settings). Beyond well-established psychosocial programs, emerging research suggests the potential benefits of genuine piano-based interventions for complex mental health conditions. It is essential to differentiate between three distinct categories of intervention: music therapy, which involves a trained therapist using musical experiences within a therapeutic relationship to address clinical goals as reported by Hartmann et al. (2023); structured piano instruction, which focuses on skill acquisition and may have incidental therapeutic benefits (Bugos et al., 2007); and multi-component psychosocial interventions, which combine psychological therapies like CBT with case management and family support (as in the GET UP PIANO trial). While the latter does not involve music, its rigorous evaluation framework provides a valuable template for designing future trials of piano-based approaches. A small number of studies have been reported only in meeting abstracts, and their findings should be regarded as preliminary and hypothesis-generating rather than conclusive. For instance, Liu (2024) presented preliminary work on combining mental nursing with piano performance as an intervention for patients with bipolar disorder, while Zhang (2025) reported initial observations on the effect of piano playing in patients with anxiety disorder. These abstracts suggest potential avenues for future research, but in the absence of full peer-reviewed publication, replication, and detailed methodology, no claims of clinical efficacy can be drawn from them. They are mentioned here to illustrate emerging interest in this area and to highlight the need for rigorous, fully powered clinical trials to substantiate these early signals.

### 3.4 Motor rehabilitation and physical recovery

The application of piano playing extends into the domain of physical rehabilitation, where it serves as a powerful, motivating tool for improving motor function and participation following neurological injury. A compelling line of evidence comes from music-supported therapy (MST) for stroke survivors, which follows the best-practice principles of stroke rehabilitation and has been proven to instigate meaningful enhancements in motor recovery post-stroke (Grau-Sánchez et al., 2020). In a landmark randomized controlled trial, Segura et al. (2024) evaluated enriched music-supported therapy for individuals with chronic stroke, demonstrating that a structured protocol combining piano training with rhythmic auditory stimulation produced significant improvements in upper-extremity function. Complementing this efficacy evidence, Grau-Sánchez et al. (2021) established the detailed eMST protocol for chronic stroke patients, while Segura et al. (2021) developed a dedicated application to support home-based enriched MST, extending therapeutic reach beyond clinical settings. These developments are grounded in a broader consensus that methodological rigor in music-based rehabilitation must be prioritized, as expert panelists at the Neuroscience and Music VII conference emphasized the need for standardized treatment definitions, control interventions, and outcome measures (Grau-Sánchez et al., 2022). At the neurophysiological level, Ghai et al. (2021) used magnetoencephalography to demonstrate that 3 weeks of intensive piano training in stroke survivors enhanced connectivity between auditory and motor network nodes in both the α and β bands, especially in the affected hemisphere, directly linking neuroplastic change to clinical motor gains. Early work by Villeneuve et al. (2014) had already shown that a 3-week piano program improved manual dexterity and upper-extremity function in chronic stroke survivors, with larger magnitudes of change associated with higher initial motor impairment. These findings are further supported by a recent meta-analysis confirming the positive therapeutic effects of music interventions in brain-damage rehabilitation (Navarro et al., 2026), and by emerging evidence that music-based approaches may also alleviate chronic pain through psychological and neurobiological mechanisms (Fernández-Dueñas et al., 2026). Taken together, this body of work positions piano-based motor rehabilitation not merely as an adjunctive activity but as an evidence-based intervention capable of reinforcing auditory-motor functional connectivity in related brain networks.

Conversely, the field of musician’s medicine offers critical insights into the rehabilitation of the piano player themselves, effectively treating musicians as “fine motor athletes.” Studies in this area illustrate a sophisticated application of sports medicine and biomechanical principles to address playing-related musculoskeletal disorders (PRMDs). For instance, Yee et al. (2002) utilized the sociological “stress process model” to predict health outcomes in pianists, identifying pain and the belief in one’s ability to control that pain as significant predictors of physical functioning. Further, case studies by Lee et al. (2005) detail the successful rehabilitation of university pianists suffering from overuse injuries, combining medical attention, alternative therapies, and technical retraining to resume active performance. This body of work highlights that effective rehabilitation requires a holistic approach, addressing not only the physical stressors through proper technique and conditioning but also the psychological mediators, such as pain attitudes and practice habits.

### 3.5 Comparative efficacy: piano versus other modalities

While this review focuses on piano-based interventions, contextualizing these findings relative to other active music-making approaches is essential for clinical decision-making. Choral singing offers distinct advantages that individual piano practice cannot replicate, particularly regarding social cohesion and verbal fluency enhancement. Särkämö et al. (2008) demonstrated that music listening enhances cognitive recovery and mood after middle cerebral artery stroke, with subsequent trials indicating that vocal music listening specifically enhances language network reorganization and memory recovery (Särkämö et al., 2008; Sihvonen et al., 2020). The NeuroMusic trial directly comparing keyboard and singing training for older adults with mild cognitive impairment found both modalities enhance verbal memory, though singing may uniquely strengthen phonemic fluency and social connectedness through ensemble participation (Wan and Schlaug, 2010). Singing also reinforces selective attention and working memory through multitasking demands (holding vocal lines, anticipating cues, tracking lyrics), with longitudinal studies indicating that choral participation builds cognitive reserve through social engagement, benefits that solitary piano practice may not fully match (Wan and Schlaug, 2010). Conversely, piano training provides superior fine motor skill acquisition and bimanual coordination, with Bugos et al. (2007) demonstrating that individualized piano instruction enhanced executive functioning and working memory in older adults, advantages likely stemming from the multimodal integration of visual, auditory, and motor systems that singing alone does not demand.

Percussion and drumming offer alternative entry points for populations with cognitive or motor limitations that might preclude piano mastery. Rhythmic auditory stimulation through drumming can improve gait and motor timing in stroke rehabilitation without the technical barriers of melodic instrument learning (Thaut et al., 1996), though piano-based motor imagery and structured keyboard training have demonstrated specific efficacy for hand dexterity and upper extremity function in chronic stroke patients (Schlaug et al., 2008). Group improvisation emphasizes social interaction and creative expression over technical skill acquisition, potentially benefiting individuals who experience performance anxiety or cognitive overload from structured notation reading; however, the cognitive demands of improvisation may exceed capabilities for those with executive function deficits. Bieleninik et al. (2017) found that improvisational music therapy for children with autism spectrum disorder showed no significant difference in primary outcomes compared to enhanced standard care, suggesting that structured approaches may be more effective for specific populations than improvisational methods.

Relative to established non-musical therapeutic approaches, piano-based interventions show promising but differential comparative efficacy. Music therapy has demonstrated noninferiority to cognitive behavioral therapy for anxiety reduction in clinical populations, with both treatments producing clinically meaningful improvements (Erkkilä et al., 2011; Archambault et al., 2022). However, direct head-to-head comparisons between piano-specific interventions and other active music modalities remain scarce; the NeuroMusic trial represents a notable exception (Wan and Schlaug, 2010). Future research must prioritize such comparative effectiveness studies to establish whether piano offers unique benefits beyond general musical engagement and to identify which populations are best served by specific modalities.

## 4 The neural orchestration of piano performance and perception

This section delves into the brain mechanisms uncovered by neuroimaging studies.

### 4.1 The auditory-motor loop

**FIGURE 3:**
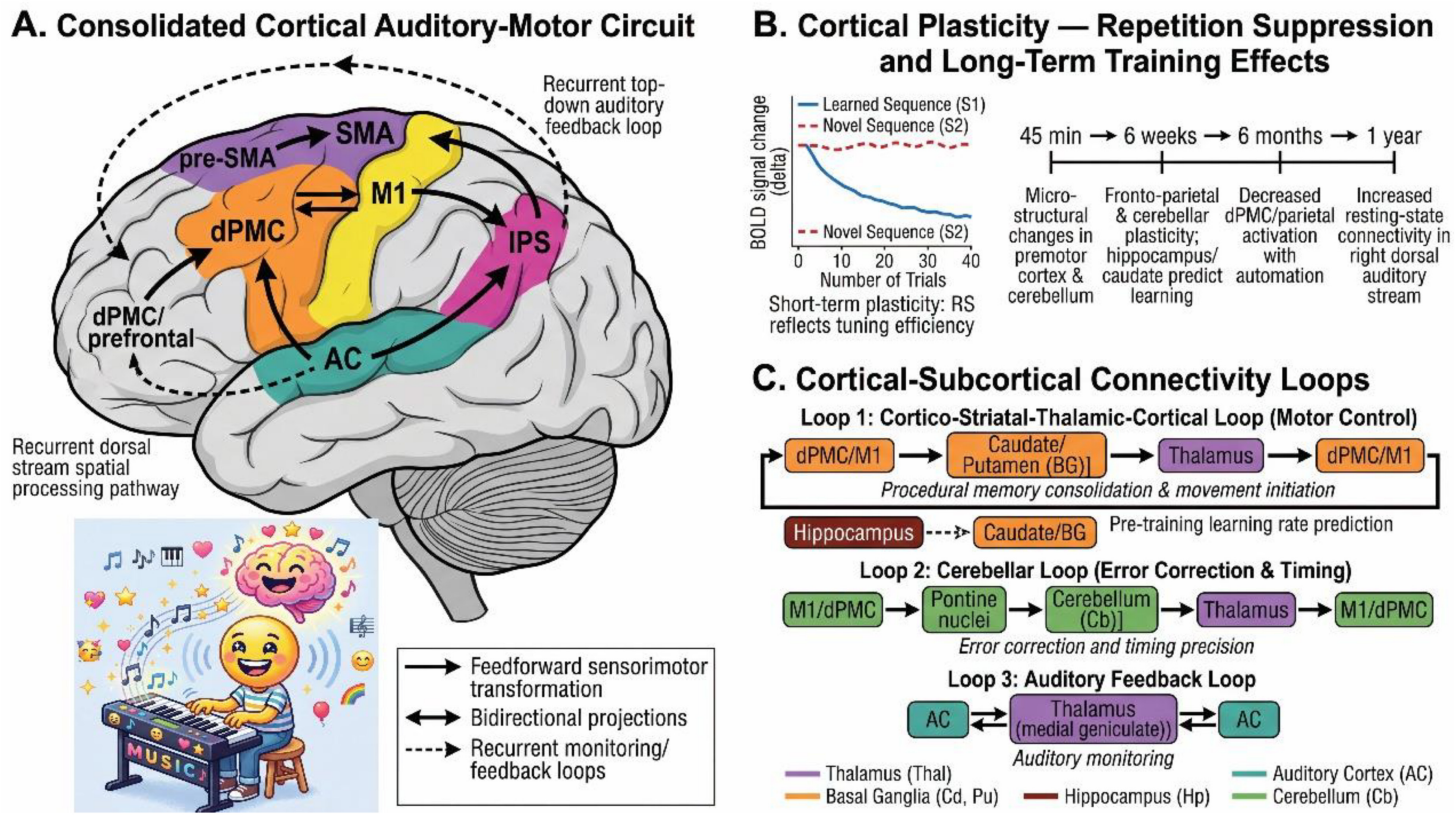
Unified neuroanatomical framework for piano performance learning and plasticity. **(A)** Consolidated cortical auditory-motor circuit. Feedforward sensorimotor transformation pathways (solid arrows) project from auditory cortex (AC, superior temporal gyrus) through the dorsal stream to dorsal premotor cortex (dPMC), pre-supplementary motor area (pre-SMA), supplementary motor area (SMA), and primary motor cortex (M1), with the intraparietal sulcus (IPS) transforming pitch information into spatial finger mappings. Recurrent auditory feedback loops (dashed arrows) include top-down projections from SMA to AC and a distinct dorsal pathway from AC to frontal motor areas supporting spatial auditory processing (Romanski et al., 1999; Rolls et al., 2023). **(B)** Behavioral and neural correlates of learning. Left: Repetition suppression (RS) for learned versus novel sequences, reflecting short-term cortical tuning. Right: Longitudinal trajectory of plasticity from brief training (45 min) to 1 year, highlighting progressive reorganization across cortical and subcortical nodes, including increased functional connectivity in the right dorsal auditory stream after extended training (Jünemann et al., 2023). **(C)** Widespread cortical-subcortical connectivity in the linked auditory and motor systems. Schematic representation of parallel processing loops: the cortico-striatal-thalamic-cortical loop (procedural memory and movement initiation), the cerebellar loop (error correction and timing precision), and thalamic auditory feedback pathways. AC, auditory cortex; BG, basal ganglia; Cb, cerebellum; Cd, caudate; dPMC, dorsal premotor cortex; GP, globus pallidus; Hp, hippocampus; IPS, intraparietal sulcus; M1, primary motor cortex; pre-SMA, pre-supplementary motor area; Pu, putamen; SMA, supplementary motor area; Thal, thalamus. The references used here are for the detailed search of the studies, this figure do not contain any copyright issues.

The exquisite coordination required for piano performance is subserved by a highly specialized auditory-motor network that seamlessly translates musical perception into precise physical action (Figure 3). This circuit encompasses the dorsal premotor cortex (dPMC), pre-supplementary motor area (pre-SMA), superior parietal cortex, and intraparietal sulcus (IPS), regions collectively engaged in mapping pitch and temporal sequences onto bimanual motor commands (Brown et al., 2013). However, this cortical scaffold does not operate in isolation. Subcortical structures, including the cerebellum, hippocampus, and caudate nucleus, are critically involved in piano learning and plasticity. Herholz et al. (2016) demonstrated that after 6 weeks of piano training, fronto-parietal and cerebellar areas showed increased responses reflecting the storage of newly learned auditory-motor associations, while pre-training activity in the hippocampus and caudate nucleus predicted subsequent individual learning rates, indicating that these subcortical regions serve as predisposition markers and plasticity hubs rather than passive participants. Tavor et al. (2020) further showed that after only 45 min of piano training, diffusion magnetic resonance imaging revealed microstructural changes in the premotor cortex and cerebellum, demonstrating that subcortical plasticity is detectable even after very brief training exposures. These findings establish that the auditory-motor loop extends well beyond cortical boundaries to include distributed subcortical nodes essential for error correction, timing precision, and procedural memory consolidation.

The functional architecture of this extended network has been delineated through repetition suppression (RS) paradigms, which exploit the principle that repeated stimulus presentation reduces neural response in dedicated cell populations, thereby indexing the tuning properties of sensorimotor circuits. During piano performance, the dPMC is crucial for planning and sensorimotor integration, the pre-SMA for sequencing and initiating motor programs, the superior parietal cortex for spatially guiding movements, and the IPS for transforming abstract pitch information often perceived in a spatial “high-low” continuum into specific finger placements on the keyboard (Brown et al., 2013). Notably, RS effects in the IPS specifically predict improvements in pitch accuracy, directly linking this region to the precision of pitch-to-motor transformation (Brown et al., 2013). This suppression pattern indicates that auditory-motor regions become more efficient, requiring less activation as sensorimotor mappings consolidate through practice, a form of use-dependent neural optimization that underlies skilled performance (Olszewska et al., 2024). Longitudinal evidence confirms that piano training induces dynamic reorganization within this network: after 6 months of training, novice pianists show decreased activation in the dPMC and parietal cortices as bimanual coordination becomes automated, reflecting a shift from spatial attention to automated movement control (Herman, 2025; Olszewska et al., 2024). At the connectivity level, Vaquero et al. (2018) demonstrated that white-matter microstructure in auditory-motor pathways predicts the rate of melody and rhythm learning, suggesting that individual differences in structural connectivity constrain training outcomes. In healthy older adults, Jünemann et al. (2023) found that 1 year of piano training increased resting-state functional connectivity in the right dorsal auditory stream, providing evidence that long-term training remodels large-scale network architecture. Martínez-Molina et al. (2021) further demonstrated that music therapy can induce resting-state network plasticity even in traumatic brain injury, highlighting the generalizability of music-driven connectivity changes across neuropathological conditions. These findings establish that the auditory-motor loop is not static but undergoes continuous refinement as a function of training-induced plasticity, enabling the fluent transformation of musical ideas into coordinated action through a dynamically optimized cortico-subcortical network.

### 4.2 Brain plasticity in experts

Long-term, intensive piano training induces profound and lasting changes in both the structure and function of the brain, a testament to the remarkable plasticity of the human nervous system. A compelling illustration of this training-induced cross-modal plasticity comes from the work of Sluming et al. (2007), who investigated orchestral musicians. They discovered that these musicians not only performed better on a visuospatial mental rotation task but also demonstrated a unique pattern of neural activation compared to non-musicians. Specifically, while both groups activated the classic visuospatial network, the musicians additionally and significantly recruited Broca’s area, a region traditionally associated with speech production and syntactic processing. The researchers interpreted this finding as the preferential recruitment of a neural circuit honed for sight-reading and motor-sequence organization to subserve a non-musical cognitive task. This suggests that the development of expert musical skills can fundamentally alter brain circuit organization, conferring a transferable cognitive advantage by repurposing domain-specific regions for broader applications.

The differences between expert and novice processing extend beyond the recruitment of additional regions to encompass more efficient and specialized neural engagement within the core sensorimotor network. This is clearly demonstrated in the fMRI study by Landau and D’Esposito (2006), which compared professional pianists to non-pianists during a sequence learning task. The results revealed a double dissociation in neural activity: the pianists showed more extensive activation throughout the task across a primarily right-lateralized network of prefrontal, sensorimotor, and parietal regions. This widespread activation reflects their long-term motor expertise (“slow learning”), providing a rich neural scaffold for performance. Concurrently, both groups showed within-session decreases in bilateral sensorimotor and parietal activation, a signature of “fast learning” and repetition suppression as a new sequence was acquired. However, the pianists’ baseline of greater and more focused activation allowed for faster reaction times and superior sequence acquisition from the outset. This indicates that the expert brain does not simply work harder; it operates with a more optimized and responsive network, capable of mobilizing extensive resources efficiently to support both the execution of well-practiced skills and the rapid acquisition of new motor sequences.

### 4.3 Neural correlates of musical emotion and imagery

The brain’s engagement with music extends beyond mere production and perception to encompass the rich, internal experiences of emotion and imagery, processes that are crucial for both therapeutic application and artistic expression. Research on emotion discrimination reveals that when the auditory system is compromised, the brain adapts by strategically reweighting the acoustic cues it uses. Hopyan et al. (2016) demonstrated this in children with cochlear implants (CIs), who, despite having limited access to fine spectral (pitch) information, could still discriminate between happy and sad music with accuracy well above chance. However, they primarily relied on tempo cues (fast vs. slow), whereas their normal-hearing peers used the more complex mode cues (major vs. minor). This finding reveals a resilient, adaptive neural strategy for emotional processing, highlighting a fundamental shift in how auditory information is prioritized in the brain when input is degraded, with implications for designing auditory interventions for the hearing impaired.

The neural basis of musical mental imagery, the “mind’s ear,” has been precisely mapped using direct neural recordings. In a landmark study, Martin et al. (2018) recorded electrocorticographic (ECoG) signals from a proficient musician who was asked to both play a piano piece with sound and then replay it while only imagining the music. By modeling the neural activity, they found that the auditory cortex was activated during mere imagery, and critically, the spectrotemporal receptive fields, the specific neural tuning for sound frequencies, were substantially similar during both perception and imagination. This provides direct evidence that internally generating a musical experience recruits the same early auditory neural populations as actually hearing it, blurring the line between perception and cognition. This framework of detailed mental simulation aligns with the concept of “enactive cognition,” which emphasizes the co-constructive role of the mind and environment. The work of Philips et al. (2024) on mentally simulating running versus “plogging” (running while picking up litter) provides a powerful model, showing that such simulations are discriminated by a broad spectrum of brain activation and are modulated by the insular cortex, a key hub for integrating internal bodily states with complex cognitive processes. This directly informs our understanding of a pianist mentally practicing a piece; it is not a vague recollection but a dynamic, whole-brain enactive simulation that engages sensory, motor, and integrative networks to reinforce learning and performance without physical movement.

## 5 The role of piano and music in special populations

### 5.1 Autism spectrum disorder (ASD)

Research into music perception within autism spectrum disorder (ASD) has revealed a striking neural dissociation that provides a powerful rationale for music-based interventions. A key study by Sharda et al. (2015) utilized a combination of functional and diffusion MRI to demonstrate that children with ASD exhibit a significant breakdown in functional connectivity between the inferior frontal gyrus (IFG) and temporal cortex during spoken-word perception, which correlated with their language abilities. However, during sung-word listening, this fronto-temporal connectivity was relatively preserved and did not differ significantly from that of typically developing controls. This finding suggests that the musical structure of song may engage neural pathways that are partially distinct from those typically recruited for speech processing, offering a potential neurobiological basis for the observation that some individuals with ASD respond positively to music-based interventions. It suggests that song can serve as an alternative conduit for engaging the language and social brain networks, offering a pathway to enhance communication and learning.

Complementing this finding are studies showing that certain musical cognitive abilities are not merely preserved but can be enhanced in ASD. Weiss et al. (2021) investigated memory for melodies and found that individuals with ASD, much like those with Williams Syndrome, displayed enhanced memory for vocal melodies compared to instrumental ones (piano or marimba). This result is noteworthy as it highlights a domain of relative strength in a population often characterized by deficits, suggesting that vocal melodies may be particularly salient or memorable. However, caution is warranted in interpreting this finding: the study reports group-level differences, and performance variability within the ASD group was not explored in detail. The fact that both ASD and Williams Syndrome groups shared this advantage, despite their divergent social phenotypes, raises intriguing questions about whether this enhanced memory stems from shared perceptual processing styles (such as heightened sensitivity to certain acoustic features) rather than from social-cognitive mechanisms.

Taken together, these studies suggest that music, and particularly vocal song, may engage auditory and cognitive pathways in individuals with ASD in ways that differ from spoken language. This provides a rationale for exploring music-based therapeutic approaches, but it does not imply that music “unlocks” latent capacities in a universal or simplistic manner. Rather, these findings point to the importance of continued research into individual differences in musical processing across the spectrum and the need for personalized, evidence-based approaches to music intervention.

### 5.2 Williams syndrome

Williams Syndrome (WS) presents a distinctive cognitive profile characterized by a pronounced affinity for music, often coupled with significant intellectual disability and language impairments. This seemingly innate attraction to music has frequently led to the assumption of superior musical abilities in this population; however, empirical evidence reveals a critical dissociation between interest and skill. Kitamura et al. (2020) demonstrated that children with WS perform significantly worse than typically developing peers on nonverbal pitch discrimination tasks using piano tones, with performance unrelated to verbal mental age, suggesting atypical development of pitch processing independent of language trajectories (Villatte et al., 2025). Neurobiological investigations have identified enhanced auditory discrimination abilities in WS as arising from interneuron hyperexcitability in the auditory cortex, providing a mechanistic basis for both preserved low-level auditory processing and atypical higher-order musical cognition. This dissociation between high motivation for musical engagement and objectively measured perceptual ability underscores the necessity of conducting thorough, individualized assessments before implementing music therapy or instructional programs. Martínez-Castilla et al. (2019) further demonstrated that musically trained individuals with WS outperform untrained counterparts in prosody discrimination, suggesting that structured musical practice can leverage genuine interest to achieve therapeutic gains provided interventions are calibrated to individual auditory profiles rather than assumed universal talent (Villatte et al., 2025). Conflating enthusiasm with expertise risks frustration and disengagement; precise characterization of specific auditory and musical strengths and weaknesses enables the design of personalized interventions that optimize therapeutic and educational outcomes.

### 5.3 Congenital amusia

The study of congenital amusia, a neurodevelopmental disorder characterized by lifelong difficulties in pitch perception, provides a unique window into the potential overlap between musical and other cognitive systems, particularly those involved in magnitude processing. Research by Nunes-Silva et al. (2016) explored this connection by investigating whether the pitch height discrimination deficits central to amusia, a form of magnitude processing, would extend to the domain of numerical magnitude. In their quasi-experimental pilot study, individuals with congenital amusia and typically developing controls performed nonsymbolic and symbolic magnitude comparison tasks, as well as a number line estimation task. The results revealed a complex dissociation: while individuals with amusia showed no significant impairments in nonsymbolic numerical magnitude discrimination or mental number line representation, they exhibited significantly worse performance on the symbolic magnitude task. This pattern suggests that the core deficit in amusia may not lie in a generalized magnitude system shared by pitch and number, but rather in a more specific disruption in accessing or processing symbolic representations. The findings propose that in addition to their well-documented pitch processing deficit, individuals with congenital amusia may have a related deficit in efficiently connecting nonsymbolic numerical representations to their symbolic counterparts (e.g., Arabic digits). This implies that the disorder could involve a broader issue with cortico-cortical connections between association areas responsible for integrating different types of abstract information, offering a new perspective on the cognitive architecture of this condition.

## 6 Toward intelligent human-computer systems: translating evidence into interaction

**FIGURE 4:**
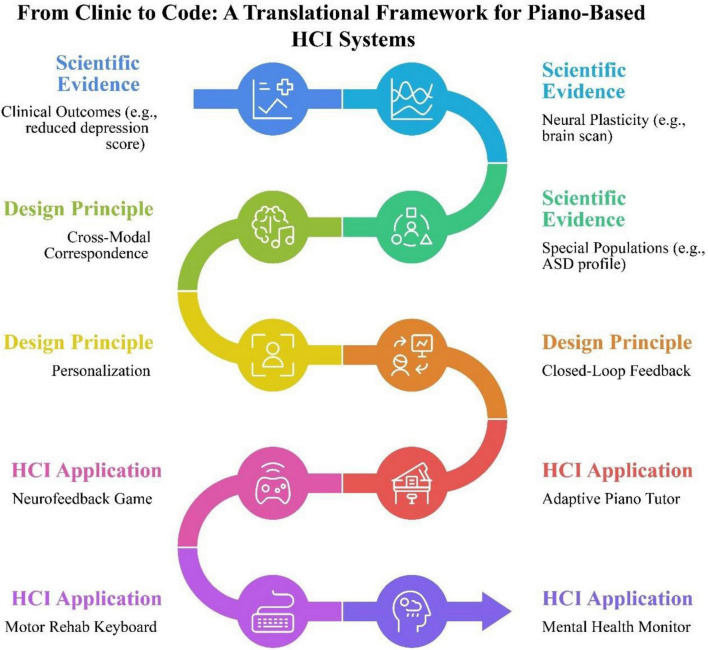
From clinic to code: a translational framework for piano-based HCI systems.

The convergence of neuroscientific evidence and clinical findings on piano interaction could provide a foundation for the next generation of intelligent human-computer systems (Figure 4). By translating principles of how the brain processes, learns from, and emotionally responds to music, researchers might design interfaces that are more intuitive, adaptive, and effective, though such applications remain largely conceptual and would require extensive validation before clinical deployment.

### 6.1 Principles for design

A key principle for designing such systems would involve leveraging the brain’s innate cross-modal correspondences. The work of Salgado-Montejo et al. (2016) empirically demonstrated that these correspondences directly influence action, showing that higher-pitched sounds consistently bias hand movements upward while lower-pitched sounds bias them downward. This finding could inform gesture-based interface design; for instance, a music production application might use vertical hand gestures to control pitch, or a rehabilitation system could employ spatially mapped sounds to guide arm movements in ways that feel natural and predictable. However, such applications remain speculative and would require rigorous user testing to validate their efficacy.

Furthermore, the move toward truly intelligent systems would necessitate a shift from one-size-fits-all approaches to deep personalization. Research by O’Toole et al. (2021) revealed that emotional responses to single musical notes are significantly modulated by individual musical proficiency, learning style, and personality traits, particularly conscientiousness. This insight suggests that future therapeutic or educational systems might incorporate user state modeling; for example, a mental wellbeing system could potentially adapt its soundscape in real-time, using complex chord progressions for musically proficient users while relying on simpler tempo adjustments for novices. Such adaptive capabilities remain theoretical, as the computational models and clinical validation required for implementation do not yet exist.

### 6.2 Applications in health-tech

The principles derived from piano-based neuroscience suggest several promising directions for future health technologies, though it must be emphasized that the systems described below represent conceptual proposals rather than established clinical tools.

**FIGURE 5:**
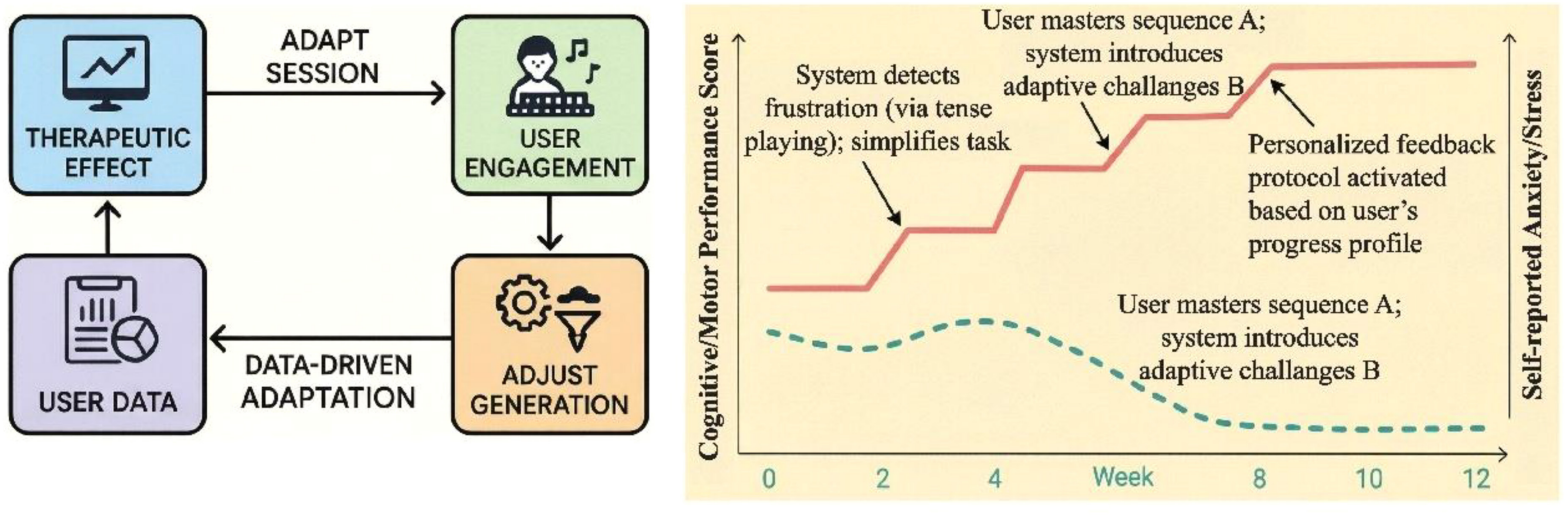
Potential impact of a personalized, closed-loop piano intervention.

One potential application lies in neuromodulation and neurofeedback. Building on Carrillo-Reid and Yuste’s (2020) description of “playing the cortex like a piano” through holographic optogenetics, one might envision bidirectional brain-computer interfaces that read real-time neural activity (e.g., via EEG) to decode brain states associated with anxiety or depression, then provide contingent auditory feedback using dynamically generated piano sounds (Sayal et al., 2025). For instance, a therapeutic target state could theoretically be mapped to harmonious melody; as neural activity approached this target, the music might remain pleasant, while deviations could produce dissonance. This closed-loop concept is grounded in neurofeedback principles, but no such system has been implemented or tested in clinical populations. Recent systematic reviews of music-based neurofeedback confirm that while the approach is theoretically promising, substantial methodological challenges remain, including high rates of non-responders and limited research on music-specific feedback interfaces (Figure 5; Sayal et al., 2025).

In motor rehabilitation, methodologies used to study musicians might be adapted for patients recovering from stroke or traumatic brain injury. Drawing on electromyography (EMG) and motion capture techniques employed by McCrary et al. (2023) to assess neuromuscular fatigue in pianists, researchers could potentially develop “smart” keyboards that monitor muscle activation to detect harmful co-contractions while analyzing ergonomic efficiency of finger movements (Wang et al., 2024). Such a system might provide real-time multimodal feedback, perhaps altering timbre when muscles are relaxed or visually highlighting inefficient movement paths. This remains a conceptual translation of research methods into therapeutic devices; no such system currently exists, and development would require substantial engineering and clinical validation.

For mental health assessment, AI-driven analysis of musical improvisation could potentially provide quantitative metrics for emotional expression. Hartmann et al. (2023) found that specific features of musical interaction in piano improvisations predicted clinical improvement in depression treatment. Building on this, one might envision an application that guides users through improvisational exercises, analyzing parameters such as tempo variability, harmonic complexity, and dynamic range to track subtle emotional shifts over time. However, reliable clinical tools based on this principle would require extensive development, including robust algorithms, normative datasets, and controlled trials to establish efficacy (Wei and He, 2026).

### 6.3 Applications in education and training

The integration of intelligent systems into music education represents another promising but largely unrealized frontier. Researchers have explored adaptive piano tutoring systems using Convolutional Neural Networks (CNNs) to analyze performance curriculum systems (Yang and Zhang, 2022). In principle, such systems might utilize computer vision to assess real-time video of students’ practice sessions, evaluating hand posture, wrist elevation, and finger curvature to provide immediate corrective feedback. However, application to nuanced piano pedagogy and validation as an effective teaching tool remain future research endeavors.

## 7 Challenges and future directions

### 7.1 Methodological heterogeneity

A significant challenge in consolidating the evidence base for piano-based interventions is the pronounced methodological heterogeneity present in the current literature (Table 2). The field comprises a mix of robust, large-scale randomized controlled trials (RCTs), such as the GET UP PIANO trial, alongside a considerable number of small-sample studies, case series (e.g., Lee et al., 2005), and preliminary findings reported only in meeting abstracts (e.g., Liu, 2024; Zhang, 2025). While these smaller and preliminary studies are valuable for identifying novel applications and generating hypotheses, their prevalence creates a fragmented evidence landscape. This heterogeneity in design, sample size, intervention protocols, and outcome measures makes it difficult to perform meaningful meta-analyses or draw definitive conclusions about efficacy across different populations. Critical gaps in quantitative reporting further complicate interpretation. Few studies report effect sizes (Cohen’s *d*, η^2^, or equivalent), and MCID thresholds are rarely referenced. For example, Song and Guan (2025) reported substantial raw score reductions on anxiety and depression scales without effect sizes or MCID context; Bugos et al. (2007) reported significant cognitive improvements without quantifying the magnitude of benefit relative to established clinically important thresholds. This absence of standardized effect metrics precludes comparison across studies and limits clinical decision-making. Future research must prioritize reporting of effect sizes, confidence intervals, and MCID-referenced interpretations to enable meaningful synthesis and application. Consequently, a paramount future direction is the implementation of more large-scale, standardized RCTs. These trials should prioritize pragmatic designs that test effectiveness in “real-world” settings, employ active control groups to account for non-specific effects of attention and engagement, and utilize consistent, validated outcome measures across studies. This rigorous approach is essential to move the field from promising pilot data to established, evidence-based practice, providing the clear, high-quality data needed to secure funding, inform clinical guidelines, and confidently integrate these interventions into standard care pathways.

**TABLE 2:** Challenges and future research directions in piano-based intervention research.

Challenge category	Description of the challenge	Proposed future research direction	Key considerations
Methodological heterogeneity	The literature is a mix of robust RCTs, small-sample studies, and preliminary meeting abstracts, making synthesis difficult.	Conduct large-scale, pragmatic RCTs with active control groups.	Standardized outcome measures across studies; focus on “real-world” effectiveness.
Mechanism elucidation	Piano interventions are complex; it is unclear which components (motor, cognitive, social) drive benefits.	Deconstruct interventions using component-analysis designs to isolate active ingredients.	Compare piano training to other complex motor/cognitive tasks; use neuroimaging to track mechanistic pathways.
Ethical HCI design	Intelligent systems handle sensitive biometric and health data, risking privacy breaches and inequitable access.	Embed “Ethics by design” principles: data anonymization, transparent algorithms, and equitable design.	Prioritize data security; ensure affordability and accessibility for diverse users, including those with disabilities.
Long-term engagement	Many digital health tools suffer from waning user interest after the initial novelty wears off.	Design for intrinsic motivation using self-determination theory (autonomy, competence, relatedness).	Implement dynamic AI that adapts to user motivation; incorporate meaningful social features; prevent monotony.

### 7.2 Mechanism elucidation

A second critical challenge lies in the need for greater mechanism elucidation. While a growing body of evidence documents the benefits of piano-based interventions for everything from executive function to depression, the fundamental question often remains: *why* does it work? A typical piano intervention is a complex package comprising multiple potential active components, including the cognitive challenge of learning notation, the fine motor skill acquisition, the auditory processing load, the potential for social interaction in group settings, and the sheer enjoyment of music-making. It is unclear whether the observed benefits are driven by one primary mechanism or the synergistic effect of all. For instance, the cognitive improvements seen in the study by Bugos et al. (2007) could be attributed to the cognitive challenge of sequence learning, the motor planning required, or a combination of both. Future research must move beyond simply documenting outcomes and begin to deconstruct these interventions. This requires carefully controlled studies that isolate variables, for example, comparing a group learning piano on a keyboard to a group learning a similarly complex video game or a group engaging in rhythmic drumming. By dissecting the “black box” of piano-based interventions, researchers can identify the core ingredients of its efficacy, allowing for the refinement and optimization of protocols to be more efficient, targeted, and personally tailored to an individual’s specific therapeutic or developmental needs.

### 7.3 Ethical HCI design

As the field advances toward intelligent human-computer systems for piano-based therapy and training, a paramount challenge that emerges is the imperative of ethical HCI design. The development of systems that leverage sensitive biometric data, such as neural activity for neurofeedback, electromyography for motor rehabilitation, or AI-driven analysis of musical improvisations for mental health assessment, raises profound ethical questions. First and foremost is the critical issue of data privacy and security. The intimate data collected, which could reveal a user’s emotional state, cognitive decline, or motor capabilities, must be protected with the highest security standards to prevent breaches and misuse. Furthermore, these technologies must be designed with accessibility and equity at their core. There is a significant risk that such advanced, and potentially costly, systems could exacerbate existing health disparities, becoming tools only for the affluent. Designers must therefore prioritize creating low-cost, scalable solutions and ensure interfaces are usable by individuals with varying levels of physical ability, technological literacy, and musical experience. Finally, the principle of user autonomy and transparency is crucial. Users must have full control over their data and a clear understanding of how algorithms are interpreting their playing to generate feedback or clinical insights. Without a robust ethical framework that prioritizes privacy, equity, and transparency, the immense therapeutic potential of these intelligent systems risks being undermined by a loss of trust and the creation of new barriers to care.

### 7.4 Long-term engagement

A final, critical challenge in the development of intelligent piano-based systems is ensuring long-term user engagement. The therapeutic and cognitive benefits documented in research, such as the improvements seen after 6 months of training in studies like Song and Guan (2025) and Bugos et al. (2007), are contingent upon consistent practice. However, many digital health and learning tools suffer from a “novelty effect,” where initial interest wanes over time. To overcome this, HCI systems must be designed to be intrinsically motivating, moving beyond simple gamification (e.g., points and badges) to create a deeply rewarding user experience. This involves leveraging principles from self-determination theory, such as fostering a sense of autonomy by allowing users to choose their musical repertoire and set personal goals, competence by providing adaptive challenges that perfectly balance difficulty with skill to maintain a state of flow, and relatedness by creating opportunities for social connection, such as virtual duets or shared performance spaces. The system’s AI, informed by studies on the temporal dynamics of engagement like those by Hartmann et al. (2023), could in principle proactively detect waning motivation, for instance, through a decline in practice frequency or rhythmic creativity and dynamically intervene by introducing new musical genres or collaborative challenges. However, such adaptive systems remain conceptual and would require substantial development and validation before they could be implemented in practice.

### 7.5 Limitations of the study

1. The narrative synthesis approach carries inherent risks of overrepresenting positive findings while negative or null results remain unpublished. Formal assessment of publication bias (e.g., funnel plots) was not feasible.
2. Our search was restricted to English-language publications, potentially excluding relevant research from East Asian and European countries where piano education and music therapy research are prevalent.
3. We did not conduct formal risk-of-bias assessment using standardized tools (e.g., Cochrane RoB, NOS, MMAT). Our synthesis includes both rigorous RCTs and preliminary meeting abstracts of varying methodological quality without systematic weighting of evidence strength.
4. Variability in intervention protocols, outcome measures, and populations precludes quantitative meta-analysis and limits conclusions about optimal intervention parameters.
5. Applications discussed in Section 5 remain largely conceptual and await empirical validation.

Despite these limitations, this review provides a comprehensive overview of current evidence on piano-based interventions, offering a foundation for future research and identifying critical gaps to advance the field.

### 7.6 Practical implications of the study

For clinicians, piano-based interventions should be matched to specific therapeutic goals: structured training for cognitive enhancement, improvisational approaches for emotional regulation. Prior to implementation, assess individual musical interest, physical capacity, and cognitive baseline rather than assuming universal suitability. Monitor for frustration or physical strain, particularly in older adults, and coordinate with multidisciplinary teams to integrate piano interventions with broader treatment plans.

For rehabilitation specialists, pianists should be treated as “fine motor athletes” with attention to ergonomic efficiency and injury prevention. Piano tasks can serve functional rehabilitation goals bimanual coordination for daily activities, rhythmic entrainment for gait improvement. Individualize equipment adaptations such as keyboard height or weighted keys, and track outcomes using validated functional measures rather than musical performance alone.

For music educators, assess actual musical capacity rather than assumed talent, particularly for students with special needs. Employ multi-sensory approaches including color-coded keys and tactile markers, maintain predictable lesson structures for students with ASD or ADHD, and break skills into manageable components. Collaborate with families and therapists to align instruction with educational and therapeutic goals.

## 8 Conclusion

This review synthesizes evidence that piano-based benefits operate through a three-tiered hierarchical mechanism: foundational sensorimotor engagement activating auditory-motor networks, cognitive-emotional mediation translating neural activation into functional gains, and contextual modulation determining individual outcomes, suggesting that benefits are not automatic but configuration-dependent. Based on this model, we propose a prioritized research agenda: first, mechanistic deconstruction through component-controlled studies isolating piano-specific effects; second, standardized metrics including core outcome sets and MCID thresholds to enable quantitative synthesis; third, rigorous RCT validation of adaptive HCI systems; and fourth, equity-focused evaluation across diverse populations. Our hierarchy of recommendations indicates strong evidence for cognitive enhancement in healthy older adults, moderate evidence for anxiety reduction and motor rehabilitation, and preliminary evidence only for severe mental illness applications and intelligent systems. Advancing this agenda, from descriptive studies to mechanistic understanding, from pilot trials to standardized research, and from conceptual promise to validated tools will position the piano as an evidence-based interface for enhancing cognition, emotional wellbeing, and physical recovery.
